# Nanofibrous Scaffolds for Diabetic Wound Healing

**DOI:** 10.3390/pharmaceutics15030986

**Published:** 2023-03-19

**Authors:** Anna Yusuf Aliyu, Oluwatoyin A. Adeleke

**Affiliations:** College of Pharmacy, Faculty of Health, Dalhousie University, Halifax, NS B3H 4R2, Canada

**Keywords:** skin regeneration, diabetic wound, diabetes, nanofibrous scaffolds, wound healing, polymeric biomaterials, tissue engineering, electrospinning, phase separation, self-assembly, wound dressings, antibiotics, phytoconstituents

## Abstract

Chronic wounds are one of the secondary health complications that develop in individuals who have poorly managed diabetes mellitus. This is often associated with delays in the wound healing process, resulting from long-term uncontrolled blood glucose levels. As such, an appropriate therapeutic approach would be maintaining blood glucose concentration within normal ranges, but this can be quite challenging to achieve. Consequently, diabetic ulcers usually require special medical care to prevent complications such as sepsis, amputation, and deformities, which often develop in these patients. Although several conventional wound dressings, such as hydrogels, gauze, films, and foams, are employed in the treatment of such chronic wounds, nanofibrous scaffolds have gained the attention of researchers because of their flexibility, ability to load a variety of bioactive compounds as single entities or combinations, and large surface area to volume ratio, which provides a biomimetic environment for cell proliferation relative to conventional dressings. Here, we present the current trends on the versatility of nanofibrous scaffolds as novel platforms for the incorporation of bioactive agents suitable for the enhancement of diabetic wound healing.

## 1. Introduction

Diabetes mellitus (DM) is a chronic condition characterized by high blood glucose level (hyperglycemia) due to the body’s inability to properly produce or use insulin. Poorly managed diabetes disease can lead to tissue and organ damage, such as in the kidneys, heart, and nerves. Although injuries can occur in different parts of the body, leg and foot ulcers are the most common wounds that occur in diabetic patients. These are generally difficult to heal and prone to infection due to decreased circulation, leading to an increased risk of needing leg amputation. Hyperglycemia can disrupt the body’s immune system, which makes diabetic patients susceptible to different kinds of infections [1,2]. Furthermore, it compromises the functions of microvascular tissues, resulting in decreased oxygen and nutrients supply to injury sites, which consequently slows down the healing process, especially in the lower extremities such as the foot [3]. It is well known that hyperglycemia increases the incidence of oxidative stress and results in the increase in inflammatory mediators, which can lead to both systemic and local inflammation. Scientific studies have shown that hyperglycemia is a major cause of oxidative stress within pancreatic cells. Therefore, hyperglycemia-induced oxidative stress can be reduced by maintaining normal glycemic levels to prevent tissue damage caused by oxidative stress [4,5,6,7].

Wound healing is generally classified into either acute or chronic based on how long the wound takes to recover. Normal wound healing occurs in four different stages, namely, hemostasis, inflammation, proliferation, and remodeling. For a wound to heal completely, it must go through these phases in the right order and expected duration [8,9]. Acute wound healing normally starts with hemostasis, which is the body’s ability to form clots immediately after an injury is detected to prevent excessive blood loss and microorganisms from entering the body through the open laceration. The second phase, inflammation, involves the body’s response to sudden injury by sending out inflammatory cells to the wounded site to initiate the healing process. This can lead to swelling, redness, heat generation, and pain in the injured area. The proliferative phase overlaps the inflammatory stage in which new tissues and blood vessels formation begin through a process known as angiogenesis. Extracellular matrix construction is also initiated to fill up the wounded area. The remodeling phase, on the other hand, is the last step in the wound healing process, and it involves the maturation of the extracellular matrices into a scar and an increase in its tensile strength. This process also leads to a reduction in the number of capillaries due to their aggregation into larger blood vessels [9,10,11].

Chronic or non-healing wounds are associated with a prolonged and generally incomplete recovery process. Non-healing wounds can occur because of different pathological conditions such as diabetes, obesity, or even stressed environmental conditions [12]. With diabetic patients, a persistent inflammatory phase lasting more than twelve weeks is observed and can lead to the obstruction of granular tissue formation. In addition, most diabetic patients suffer from peripheral neuropathy (i.e., nerve damage that can lead to a complete lack of sensation), which can make external wounds, such as cuts or burns, go unnoticed by affected individuals, since they are unable to feel any pain or discomfort. This can increase the chances of infection if the injury is not properly cared for on time. Therefore, many complications can arise because of non-healing wounds in diabetic patients, such as difficulty in walking, gangrene, osteomyelitis, abscess, cellulitis, etc. Although the direct link between impaired wound healing and the pathophysiology of diabetes is still unknown, there are some molecular factors that have been associated with enhancing this process (e.g., vascular endothelial and platelet-derived growth factors) in diabetic rats [13].

Nanofibrous scaffolds are artificial extracellular matrices that mimic the natural environment for tissue formation. This type of scaffold is more advantageous than other available variants because of its large surface-to-volume ratio, which leads to the efficient promotion of cell adhesion, proliferation, and differentiation. They are made up of either natural or synthetic polymers through different techniques, which generally include phase separation, self-assembly, melt blowing, and electrospinning [14,15,16,17]. The electrospinning technique has been found to be more useful in the fabrication of nanofibrous scaffolds for wound healing, drug delivery, and tissue engineering purposes. Nanofibrous scaffolds have shown promising results in wound healing because they protect the injured area from moisture loss, aid the removal of exudates, and inhibit the incursion/growth of microorganisms. These special properties of nanofibrous scaffolds promote faster healing of wounds as compared to other conventional dressings, especially in chronic wounds, such as diabetic ulcers [16,17].

Researchers continue to explore novel wound dressing techniques that can optimally prevent microbial infestation in diabetic lesions to reduce the risk of sepsis and enhance the healing progression, such that full anatomical and physiological functions are regained, and complications that can result in amputation or mortality are avoided. Nanofibrous scaffolds have shown promising outcomes in the management of open wounds, and their nature allows for the possibility of incorporation of therapeutics, which can be administered topically, thereby reducing the possible side effects in non-target cells while increasing the localized bioavailability of these drugs at the wounded site [13]. Different strategies have been developed for the treatment of chronic wounds, such as autologous skin grafting, which is the most clinically successful strategy due to its non-immunogenic nature. It, however, cannot be used in large, injured regions exceeding sixty percent of the patient’s total body surface area, which makes the need for alternative approaches to accelerate the process of chronic wound healing an important one. Scaffolds mimic the extracellular matrix and can serve as an appropriate microstructure for native cell proliferation, migration, and differentiation. Moreover, incorporating nanoparticles, such as zinc oxide, into nanofibrous scaffolds can stimulate angiogenesis, which can promote faster wound healing [18].

This compilation appraises scholarly work focused on the applications of nanofibrous scaffolds in wound healing and skin regeneration specifically for diabetic lesions. Besides, their ability to effectively encapsulate therapeutic agents, such as antimicrobials, anti-inflammatory agents, and healing enhancers that prevent infection at the wound site and aid the overall curative process of chronic wounds, will be covered. It also provides some insight into conventional diabetic wound management approaches, highlights basic contrasts between the use of orthodox dressings and nanofibrous scaffolds, as well as recent advancements in the fabrication of these scaffolds. This review was executed based on extensive evaluations of scientific articles published over the past decade or so, which are centered on recent developments in the use of nanofibrous scaffolds for facilitating the healing of diabetic wounds via Google Scholar, ResearchGate, Science Direct, Scopus, and Pub Med. This initial search yielded two hundred and ninety-four scientific articles, which were then narrowed down to just over one hundred peer-reviewed publications centered on the title of this paper or closely related to the subject matter employing keywords such as “nanofibrous scaffolds”, “diabetic wounds”, “diabetes mellitus”, “chronic wounds”, “wound healing”, and electrospun nanofibers”.

## 2. Conventional Approaches Employed for Treating Diabetic Wounds

The standard treatment for diabetic wounds involves the management of the underlying cause of the wound, control of ischemia, infection control, wound debridement, offloading to relieve pressure (in diabetic foot ulcers), and wound dressing. The primary aim of diabetic wound dressings is to create and maintain a moist and microbe-free environment for the wound. It is also important that the dressing should prevent further trauma, promote granulation, and absorb chronic wound fluids [19].

There are different types of wound care approaches among which surgical and autolytic debridement are the most employed in diabetic ulcers. Autolytic debridement is the most conservative and highly selective option, which involves the use of phagocytic cells and proteolytic enzymes to remove necrotic tissues. The main limitation of this method is its ineffectiveness in the treatment of infected diabetic wounds. Surgical or sharp debridement has proved to be more effective than the autolytic method. This procedure is a fast and selective process, and it is usually carried out under anesthesia either in an operation theatre or bedside, depending on the amount or size of necrotic tissues that is to be removed until tissue viability is regained [20,21,22].

Wound care and dressing are an essential part of treating diabetic ulcers, and there are various topical agents, such as hydrocolloids, hydrogels, alginates, silver-impregnated dressings, and devices such as hyperbaric oxygen therapy and vacuum-aided gadgets, which are used for this purpose [23,24]. There is no single dressing that possesses the entire requirements of a diabetic foot ulcer patient; therefore, it is important to select a dressing which will be convenient for each patient and meet their needs. For instance, foam and alginate dressings are suitable for highly exuding wounds due to their absorbent nature, and non-adhesive dressings are well accepted because they are simple and inexpensive, and silver-impregnated dressings are used in infected lesions, while occlusive dressings should be avoided. Dressings should be carefully selected based on the characteristics of the ulcer, patient requirement, and cost [24,25,26].

Offloading is another fundamental approach used for the management of diabetic ulcers, and it refers to the act of relieving pressure from an ulcerated area. It is also used to describe the redistribution, decrease, and removal of injurious force applied to the wound site. Although it is not possible to completely remove the pressure on the injured area, there are devices that help reduce the duration and magnitude of the force. Devices used can either be cast or non-cast offloading devices. When using casts, it is important to always keep it dry and continuously check out for developing sores, malodour, and any abnormalities around the wound location. Non-cast devices include healing sandals, crutches, wheelchairs, etc. Offloading devices should be selected based on the patient’s needs [27].

The treatment of active infection, as well as glycemic control, is also an essential part of wound treatment. It is an established fact that infection is the predictor of slow wound healing, thus it is imperative to appropriately treat with antibiotics as soon as an infection is recognized or as preventive therapy before microbial growth begins. The antibiotic therapy should be targeted at Gram-negative cocci moderate infections for one to two weeks, while broad-spectrum antibiotics should be administered for two to three weeks in severe infections. It is a general recommendation for diabetic patients to keep track of their blood glucose level and adopt lifestyle changes that will help maintain a glucose level within the normal range. Several studies have shown the positive impact of glycemic control and wound healing and, there is a thirty-five percent decrease in lower extremity amputation in type 2 diabetic patients with intensive glycemic control. Therefore, it is important to keep both microbes and glucose level abnormalities in diabetic patients under control for effective wound healing [21,26].

## 3. Polymers Used in the Fabrication of Nanofibrous Scaffolds

There are two broad categories of polymeric materials used in the construction of nanofibrous scaffolds for diabetic wounds. These include synthetic polymers, which are manmade, and natural polymers that exist in nature. Polymers are used in the manufacture of many products that people utilize in everyday life [28]. The polymeric materials used in the manufacture of nanofibrous scaffolds are carefully selected based on the cellular environment and interactions needed for the specific application, thus scaffold biomaterial can either be synthetic or of natural origin, biodegradable, or non-biodegradable [29]. Several nanofibrous scaffolds, for wound healing applications, have been developed from a wide range of synthetic and natural polymers, which are either easily degraded in human beings or are biostable (Table 1). Biodegradable polymers are more acceptable for fabricating scaffolds for skin tissue engineering, while non-biodegradable polymers are suitable as wound dressings for delivering cells (e.g., stem cells, fibroblasts) and other bioactive materials into wounds to accelerate healing [30].

### 3.1. Natural Polymers

A wide range of natural polymers, such as chitosan, cellulose, gelatin, collagen, poly (amino acids), hyaluronic acid, etc. have been processed and assessed for their skin-repairing abilities, and they each have specific characteristics that promote diabetic wound healing.

#### 3.1.1. Chitosan

Chitosan is derived from chitin, but, due to the insoluble nature of chitin in aqueous solutions, it is converted into chitosan by thermochemical deacetylation. The physical and chemical properties of chitosan are attributed to its degree of deacetylation and molecular weight. Chitosan possesses antibacterial properties, film-foaming qualities, strong wound adhesive characteristics, and promotes blood coagulation for the enhancement of wound healing [15,50]. It is difficult to electrospin due to its poor solubility in most of the widely used organic solvents and needs to be blended with other natural or synthetic polymers, such as polyvinyl alcohol and gelatin, to improve its solubility and make it more spinnable [15,50,51,52].

#### 3.1.2. Collagen and Gelatin

Collagen is the most extensively distributed type of protein in the human body. It is widely used in tissue engineering as a biomaterial because of its biodegradable, biocompatible, and versatile nature. It is, however, difficult to sterilize it without altering its chemical structure because it is a protein [53]. Gelatin is derived from collagen, which makes its chemical constituents like that of collagen and a good alternative to collagen. Gelatin has been used either as a single entity or in combination with other polymers to fabricate nanofibrous scaffolds of high porosity, extensive surface area, and well-connected pores, making them good candidates in tissue engineering and diabetic wound management [54,55].

#### 3.1.3. Hyaluronic Acid

Hyaluronic acid is a polysaccharide polymer that forms one of the major constituents of the extracellular matrix and significantly influences cell migration and proliferation [56]. It has been applied as dermal fillers, scaffolds in tissue engineering, and diabetic wound healing. Its insolubility in organic solvents is a major disadvantage associated with its use [57]. Hyaluronic acid (low and medium molecular weight variants)-based dressings can serve as lubricants, possess good water absorption properties, enhance the deposition of collagen at the injury site, and, overall, improve epithelial migration and angiogenesis, hence facilitating wound closure. However, the high molecular weight hyaluronic acids interfere with the supply of nutrients to the wound site and therefore hinder the wound healing process, making this category an unsuitable choice for such applications. [58].

#### 3.1.4. Cellulose

Cellulose is a biodegradable carbohydrate found predominantly in plants. It possesses a non-toxic nature, impressive mechanical strength, and biocompatible nature. Cellulose acetate, an improved version of cellulose, produces nanofibers with better elasticity and stability [57]. Although cellulose is insoluble in most solvents, it is highly stable in the liquid state and possesses good optical and mechanical characteristics. It can retain moisture, which makes it useful in wound treatment [59]. Carboxymethyl cellulose, a derivative of cellulose, is widely reported to have the potential to heal wounds due to its cell compatibility, ability to form a matrix, and cross-linking capabilities. Though there is limited information on the use of carboxymethyl cellulose alone in wound healing, growth factors have been incorporated into it for the treatment of diabetic wounds in streptozotocin-induced diabetic rats, and it resulted in increased migration and proliferation of cells [60].

#### 3.1.5. Poly (Amino Acids)

Poly (amino acids) are naturally occurring biodegradable polymers and are made up of amino acid monomers, which are linked through an amide bond. They are immunogenic in nature, possess poor mechanical strength, and are soluble in acidic solvents. Poly (amino acids) based nanofibrous scaffolds, such as poly-aspartic acid, poly-lysine, poly-arginine etc., often blended with other polymeric biomaterials, have been studied and shown to have good biocompatibility and improve diabetic wound healing [61,62,63].

#### 3.1.6. Starch

Starch is a carbohydrate type of polymer that is explored in different biomedical applications, such as wound healing due to its biocompatibility, availability, and low cost. It has also been used for cell adhesion, proliferation, and regeneration, hence starch-based scaffolds have great therapeutic significance in wound healing applications [64,65]. Some undesirable properties of starch, such as its sensitivity to moisture and mechanical strength negatively, impact its application in tissue engineering, however, polyvinyl alcohol can be used as a plasticizer in its electrospinning process. The crosslinked nanofibrous scaffold of starch and polyvinyl alcohol produced a thicker nanofiber that allowed the proliferation of skin cells and, thus, could be employed in diabetic wound healing [66].

### 3.2. Synthetic Polymers

Synthetic polymers have also been processed into scaffold matrices and assessed for their wound-healing abilities. The most widely used for this purpose are polycaprolactone, polyurethane, poly lactic-co-glycolic acid, and polylactide. Although all these polymers can be processed using different methods, such as electrospinning and utilized individually, there are some limitations that they each have, which make them unsuitable for scaffolding as single entities. Thus, cross-linking two or more polymers, as well as incorporating suitable biomaterials, can improve the physicochemical, physicomechanical, and therapeutic properties of the resultant scaffolds [51,67]. For instance, polycaprolactone is a Food and Drug Administration (FDA)-approved synthetic polymer that is biocompatible, non-toxic, biodegradable, and can be easily processed into different shapes and sizes, which makes it suitable for the treatment of chronic wounds, such as diabetic foot ulcers. It, however, has a poor antimicrobial property, and, therefore, needs to be incorporated with antimicrobial agents, such as silver nanoparticles, to prevent microbial infestation [68].

#### 3.2.1. Polycaprolactone

It is an aliphatic polyester, which is slowly degradable and soluble in different kinds of solvents, thereby making it suitable to be combined with other types of polymers. Polycaprolactone is less hydrophilic than poly-lactide acid and, as such, shows less cell adhesion, thus it is often crosslinked with other polymers or bioactive agents. Polycaprolactone has been used in combination with natural polymers, such as collagen or synthetic materials such as polyvinyl alcohol, to form a nanofibrous scaffold loaded with a bioactive agent for skin regeneration purposes and promotion of diabetic wound healing [30,48,51,69,70].

#### 3.2.2. Polylactide Acid

It is a widely used polymer in nanofibers fabrication, which is obtained by polymerization of lactide for diabetic wound healing purposes [71]. It is usually crosslinked with other polymers, such as gelatin, to enhance its cell adhesion properties, or it is combined with biomaterials such as silver nanoparticles for wound dressing purposes to curb microbial growth in infected wounds [30]. It has poor compatibility when in contact with blood but combining it with chitosan or chitosan derivatives improves blood compatibility and can promote cell attachment and proliferation [72].

#### 3.2.3. Poly(lactide-co-glycolic) Acid

This is a copolymer of polylactide and polyglycolic acid that is also biodegradable and is used in controlled drug delivery and tissue engineering and has also been explored as a scaffolding matrix for bioactive molecules employed in the management of diabetic wounds [32,35]. It can be blended with various polymers as well as nanoparticles to produce an improved application [57]. Nanofibrous scaffolds consisting of PLGA, and cellulose nanocrystals impregnated with neurotensin, enhanced the healing of a full-thickness skin wound in diabetic rat [73].

## 4. Commonly Employed Manufacturing Techniques

Several methods have been employed for the development of nanofibrous scaffolds for diabetic wounds. These include electrospinning, phase separation, self-assembly, melt blowing, and templating systems [16], with self-assembly, electrospinning, and phase separation being the most utilized [14,74].

### 4.1. Electrospinning

This technique produces nanofibers from a solution of polymer(s) through an electrohydrodynamic process governed by voltage. The most common setup for this technique includes three main components, which are a high-voltage power source, reservoir (for keeping the solution), needle and syringe and collector [75]. The electrospinning process has several advantages over other methods of nanofiber fabrication because it is cost-effective, easily scalable, and a relatively simple method [55]. It produces optimally porous and flexible scaffolds with excellent moisture-absorbing characteristics, enhanced oxygen exchange qualities, and a level of antibacterial effect [72]. It can be applied in numerous fields, but it has gained popularity in the medical field specifically due to its benefits in the formulation of nanofibrous scaffolds used in skin regeneration, wound healing, and drug delivery systems [69,76].

### 4.2. Phase Separation

It is a simple method that can be used for processing either synthetic or naturally occurring polymeric materials. It involves utilizing the differences in the solubilities of two or more polymers, which allows distribution into their respective solvent systems. Thereafter, each separated polymer can be molded into structured, interwoven nanofibrous scaffold platforms for different applications (e.g., wound dressing) [77,78]. The phase separation method is categorized into the thermally induced and diffusion-induced processes. The thermally-induced process is the most widely used one, and it involves a temperature decrease as the polymer separates out of the solvent, while the diffusion induced procedure entails the immersion of the polymer-solvent mix in an antisolvent bath that partitions the solvent, allowing the polymer to separate out. By changing production parameters (e.g., polymer concentration, temperature, solvent type etc.), phase separation allows more control over scaffold thickness and porosity, which are important factors that can significantly influence their performance and application. Additionally, this method is useful for fabricating scaffolds with varying shapes as required and can easily maintain batch-to-batch consistency [79]. Besides, phase separation techniques have found use in the manufacture of vascular grafts, which are tube-like scaffolded structures typically used for redirecting movement from an area with normal blood flow to another area of the body with abnormal blood flow by reconnecting blood vessels. These grafts are useful for treating peripheral vascular disease experienced by diabetic patients [78,79,80]. Phase separation is more of a laboratory scale procedure and can only be applied to a limited number of polymers [77].

### 4.3. Self-Assembly

This involves the building of desirable nano-dimensional patterned structures through the organization of molecular building blocks that exhibit suitable intermolecular interactions and parallel control of the solution pathway [81]. These interactions are often activated through the blending of different components or by externally applied triggers, such as temperature, acid-base levels (pH), etc. The bonds formed between the molecules are non-covalent bonds such as ionic bond, van der Waals force, etc. Although these bonds are weak, their interactions produce chemically and structurally stable assemblies, which can be employed in the formation of nano-dimensional fibrous scaffolds that can optimally mimic the extracellular matrix [82,83]. Peptides are widely used in the self-assembly of nanofibers employed in tissue engineering although synthetic polymer have also been applied. Peptide amphiphiles (compounds consisting of a dialkyl head and a peptide tail) can be engineered into nanofibers with cells easily encapsulated into their structure when added during the self-assembly processes. This is unlike other fabrication approaches that often need technologically advanced instruments. The development of heparin mimetic peptide amphiphiles gels has been found to promote angiogenesis, re-epithelialization, and inflammatory responses in diabetic mice, which are essential stages in wound healing. Some drawbacks of this method are that it typically forms mechanically weak fibrous matrices, which, upon fragmentation, can pose the risk of endocytosis and can be expensive, which limits its use in regenerative medicine and tissue engineering [82,83,84,85].

### 4.4. Melt Blowing

This is a distinctive way of fabricating both micro- and nanofibers and it involves the extrusion of molten polymer through orifices that are circulated by hot, high-velocity air to produce very fine fibers that cool down and solidify under ambient conditions [78]. The system is made up of an extruder, which consists of three zones, the feeder, transition, and metering zones. This technique does not require solvents or devices operating at high voltages, and it is flexible and easy to industrialize. The production rate of the fibers is high, and their dimensions, densities, and orientations can be altered by simply changing the capillary sizes and numbers of the dies, as well as the airflow velocity. Polypropylene is one the most used polymers in melt blowing technique, but it is widely employed in bone tissue engineering and sutures. Polylactide could be utilized as a substitute for polypropylene in skin tissue engineering for the purposes of treating chronic wounds, such as diabetic foot ulcers [86,87].

### 4.5. Templating System

In this method, the biomaterial under construction into nanofibers (e.g., polymers) is synthesized inside the holes of a template membrane with uniformly sized orifices. This technique allows the synthesis of nanofibers with varying diameters, and this is usually achieved by changing the template settings [88]. The fabrication of nanofibrous scaffolds for skin tissue engineering using a template-assisted electrospinning technique was employed in the production of structural scaffolds with the use of polycaprolactone. This resulted in the increment of fibroblast cell proliferation, elongation, and enhanced wound closure rate [89].

## 5. Current Trends in the Development of Nanofibrous Scaffolds for Diabetic Wounds

**Figure 1:**
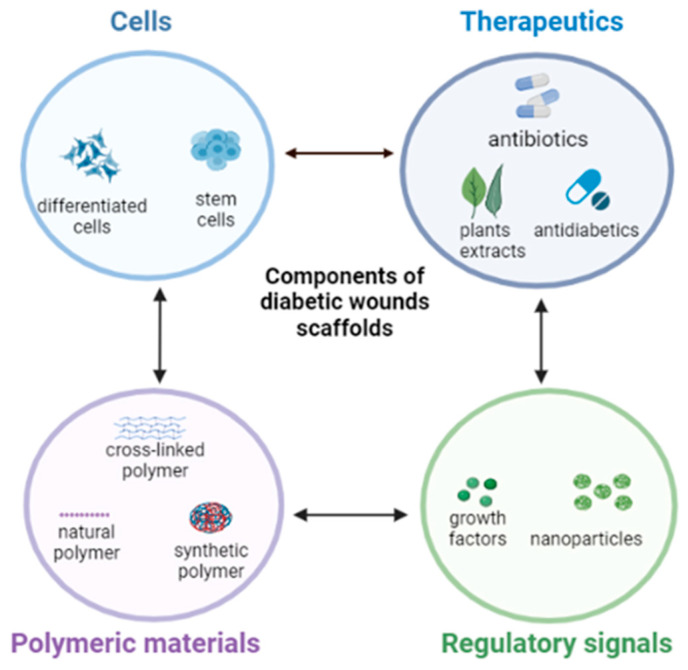
Typical bioactive components of nanofibrous scaffolds for diabetic wound healing. Create in BioRender.com.

The incorporation of various bioactive ingredients into polymer-based dressings, especially for diabetics, has shown promising results in wound care and healing. These bioactive agents include, but are not limited to, antibiotics, phytoconstituents, antioxidants, anti-inflammatory, stem cells, or growth factors (GF) [35] (Figure 1).

### 5.1. Antibiotics

Research has been channeled toward the engineering of antibiotic-loaded nanofibrous scaffolds for wound dressing purposes, such as in diabetic wounds. Such matrices allow localized wound therapy, which turns out to be more selective, effective, and minimizes adverse effects associated with systemic absorption [45]. Classes of antibiotics that have been applied for this purpose include aminoglycosides, beta-lactams, quinolones, sulphonamides, tetracyclines, etc. [45,90].

For instance, Jafari and co-workers [35] designed polycaprolactone and gelatin-based bilayered nanofibrous scaffold containing amoxicillin and zinc oxide that prolonged antibacterial effect, quickened wound contraction, elevated collagen deposition and angiogenesis and scar prevention in chronic, full-thickness diabetes wounds. Doxycycline, a broad-spectrum antibiotic, was encapsulated into a polylactide-based nanofiber specifically for the management of chronic wounds. Doxycycline release from the nanofiber was initially rapid and transitioned to a sustained release kinetics at high concentration for two weeks. It also showed a high antibacterial activity, and it inhibited the growth of *Escherichia coli* and *Staphylococcus aureus,* which indicates that it is a good candidate for the treatment of infected diabetic lesions [91].

Additionally, silver nanoparticles are widely employed in nanofibrous scaffold fabrication due to their antimicrobial property. It has bactericidal effect with decreased ability to cause systemic toxicity. Unlike other antibiotics, it prevents the development of bacterial resistance. Silver nanoparticles can also be combined with antibiotics such as sulphanilamide for synergistic antibacterial (against a wide range of both Gram-positive and Gram-negative bacteria) and wound healing effects [92,93,94]. Other than silver nanoparticles, there are several metal ions (e.g., iron, zinc, titanium, gold, copper, etc.) that possess antibacterial, tissue regeneration, and wound healing properties when fabricated as nanostructures with polymeric biomaterials for diabetic foot ulcers [31,95,96]. Cai and colleagues [38] have also reported ferrous oxide loaded onto a gelatin and chitosan nanofiber matrix to form a strong nanofibrous dressing with good antibacterial efficacy for potential diabetic wound dressing application. Another study by Lee et al. [32] fabricated coaxial sheath-core nanofibrous poly(lactide-co-glycolide) scaffold sustained the release of vancomycin and gentamicin and sped up the process of healing and repairing early-stage infected diabetic wounds.

### 5.2. Herbs and Phytochemicals

Several medicinal plant extracts are being used in the development of scaffolds for diabetic wound dressing, owing to their natural ability to fight off bacteria, act as antioxidant and anti-inflammatory effects with lower toxicity and side effects, low cost, and easy availability [97]. Asiaticoside is a phytochemical that possesses numerous therapeutic activities, such as antioxidant, anti-inflammatory, and a potential chronic wound healing ability. Silk-based nanofibrous scaffolds loaded with asiaticoside enhanced the healing of lesions on diabetic induced rat models, and it also exhibited antibacterial effects against *Pseudomonas aeruginosa* and *Staphylococcus aureus* [48]. Curcumin is another phytochemical that has strong antioxidant, anti-inflammatory, and anti- infective characteristics [98]. Polycaprolactone-based nanofiber loaded with curcumin demonstrated antioxidant and anti-inflammatory effects in diabetic mouse models as compared to nanofibers of polycaprolactone alone [33]. In vivo wound closure experiment performed on diabetic rats treated with curcumin-loaded nanofibers showed accelerated healing, and the lesion was completely closed on day fifteen, while the control group showed less than thirty percent closure at the same time point [93].

Aloe Vera gel (*Aloe barbadensis miller*) is another well-known plant chemical known for its therapeutic use in the treatment of burn wounds. It also possesses antidiabetic, anti-inflammatory, and wound-healing abilities by stimulating fibroblast and collagen synthesis to enhance lesion recovery. Aloe Vera gel incorporated into gelatin/polycaprolactone-based nanofiber scaffold was reported to have increased fibroblast proliferation, and it provided antibacterial activity and biodegradability as compared to gelatin/polycaprolactone alone [46,99,100]. Another widely recognized phytochemical known to have potent antioxidant antidiabetic and wound healing properties, which can be beneficial in the treatment of diabetic wounds, is Fenugreek. It was electrospun with silk fibroin and was found to improve collagen deposition at the injured site as well as complete re-epithelialization of the wounded area in a rat model [41,97]. Selvaraj and colleagues [42] further explored Fenugreek extract by incorporating it into a collagen/silk fibroin composite matrix, and they found that this nanofibrous scaffold had antioxidant properties, produced good biocompatibility, and aided fibroblast migration and wound closure through minimal inflammation and early epithelialization. The wound healing efficacy of polyvinyl alcohol/sodium alginate blended nanofibrous scaffolded mats containing *Calendula officinalis* extract were prepared by electrospinning and tested in male Wistar rat models. Experimental outcomes showed that the scaffolds were biocompatible, and they supported cell attachment and proliferation and injury closure [40].

### 5.3. Stem Cells

The localized administration of stem cells to open diabetic wounds through nanofibrous scaffold matrices could be a good approach for the enhancement of wound healing due to their ability to secrete immunomodulatory, anti-inflammatory, and angiogenic factors. Although different types of stem cells have been studied, mesenchymal stromal cells (MSC) gained popularity because of their therapeutic use in managing delayed wound healing. MSCs are considered “ideal cell sources for regenerative therapy with no ethical issues” and have shown significant efficacy in the healing of diabetic ulcers. Research has revealed that MSC transplantation can reduce wound dimensions, restore desirable clinical parameters, improve painless walking, and avert amputation related the diabetic foot ulcers [101].

A three-dimensional scaffold using polycaprolactone, gelatin, and pluronic-F-127 to administer bone marrow-derived mesenchymal stromal cell (BM-MSC) was developed and was seen to enhance granular tissue formation, angiogenesis, and increased collagen deposition at the wound site in diabetic mouse model [49]. Adipose-derived stem cells (ASC) are readily available, possess similar physical and functional characteristics with BM-MSC, and promote diabetic wound healing by increasing tissue regeneration and angiogenesis. It was also reported that ACS promotes cell development by depositing growth factors, such as vascular endothelial growth factor and human growth factor when used topically [102]. Fu et al. [103] noted the challenges associated with efficiently stabilizing MSC for topical administration due to the high level of proteolysis occurring at the delivery site and therefore engineered a scaffold based on reduced graphene oxide (RGO) nanoparticle combined with an acellular dermal matrix (ADM) that encapsulated MSC. The ADM-RGO scaffold matrix promoted stem cell adhesion and proliferation and was highly stable and mechanically robust. It supported excellent vascularization, collagen deposition, and fast re-epithelization on streptozotocin induced diabetic mice model, presenting a promising therapeutic approach for non-healing diabetic wounds.

In general, there are limited clinical trials reported on the use of MSCs for diabetic wound healing. A few studies on human volunteers, which used BM-MSCs based treatments, were documented, and these continue to serve as progressive evidence demonstrating the efficacy of MSCs in treating diabetic ulcers. Mainly, the injection of autologous transplantation of BM-MSC delivered by intramuscular injection or transplantation [104,105], directly on wound site [106,107], by injection into the ischemic limb Procházka et al. [108] or via the transfemoral route [109] on type 2 diabetic patients with critical limb ischemia and foot ulcers showed significant healing rate with notable improvement in walking (no discomfort), decrease in wound size and healing time, sufficient improvement in leg perfusion and vascularity of skin surrounding wound, increased oxygen pressure, as well as decreased weakness, numbness, and amputation risks.

### 5.4. Growth Factors

These are referred to as physiologically active proteins, which are involved in the proliferation, migration differentiation, and metabolism of cells. Together with cytokines, they regulate the healing process that occurs in the body. Nanoparticles loaded with either one or more growth factors showed faster wound healing because growth factors typically promote angiogenesis, inflammatory response, and remodeling. However, because of diabetes, the systemic availability of growth factors decreases [110]. Epidermal growth factor (EGF), the most studied growth factor in wound healing, stimulates cell proliferation and differentiation, and a decrease in its concentration has been linked to diabetes mellitus, which is considered one of the factors that contribute to the impaired healing process. Thus, delivering EGF by encapsulating it in suitable polymers, such as collagen hyaluronic acid composite, polyurethane and silk fibroin, has been reported to exhibit anti-inflammatory activity, which further improved wound healing in diabetic rats. Additionally, clinical studies involving the use of EGF incorporated in nano-silver scaffolded dressings displayed a significantly shorter wound repair time and increased granulation tissue in patients with diabetic foot ulcers [47,111]. Another growth factor that is widely considered for chronic wound treatment is the vascular endothelial growth factor (VEGF) due to its vasculogenic and angiogenic activity. It stimulated cell proliferation, migration of fibroblasts, deposition of collagen, and re-epithelialization when administered through scaffolds in diabetic rats [102]. Vijayan and others [43] also reported on the construction of nano-encapsulated vascular endothelial growth factor and basic fibroblast growth factors adsorbed onto electrospun collagen/PLGA/chitosan-based scaffolding structures that aided angiogenesis, cell proliferation, collagen deposition, and re-epithelialization at the diabetic wound site. The basic fibroblast growth factor plays a key role in the diabetic wound healing processes, facilitates fibroblast proliferation and neovascularization, and has anti-scaring qualities [43].

### 5.5. Anti-Inflammatory and Antioxidants

Hyperglycemia promotes the assemblage of reactive oxygen species (ROS) intracellularly, which induces oxidative stress, although oxidative stress is required for wound disinfection and boosts wound healing, and uncontrolled oxidative stress deregulates inflammation and plays a crucial part in the pathogenesis of chronic wounds. Therefore, administering antioxidants help regulates the balance of ROS in the cells [112]. Similarly, diabetic patients are more likely to experience microbial-induced inflammation due to skin injuries. Thus, anti-inflammatory agents can be used to prevent and treat that. Glutathione has both antioxidant and anti-inflammatory properties and can be utilized in scaffolds to neutralize excess ROS, as well as to prevent microbial-induced inflammation. Polycaprolactone nanofiber was attached to glutathione soaked in glutaraldehyde solution, which produced a biocompatible and biodegradable characteristic. The outcome shows a promising result that the use of glutathione-polycaprolactone nanofiber could be used for its antioxidant, anti-inflammatory, and possible antibacterial effect due to the presence of glutaraldehyde in the diabetic wound nanofiber-based therapy [39].

### 5.6. Antidiabetic Agents

Some hypoglycemic agents have been shown to reduce inflammation, a quality that can significantly speed up the healing process of diabetic ulcers and improved therapeutic outcomes. Some examples of antidiabetic agents identified in the literature to have demonstrated moderate to high-level anti-inflammatory activity include sulfonylureas, thiazolidinediones, dipeptidyl peptidase-4 inhibitors, and metformin, which is a biguanide [34,113]. In a study conducted by Cam and coworkers [113], a combination of oral hypoglycemic drugs, namely, pioglitazone, metformin, and glibenclamide, were embedded within a chitosan/gelatin/polycaprolactone and polyvinyl pyrrolidone composite nanofibrous scaffolds and assessed for their diabetic wound healing effect. This combined therapeutic system quickened diabetic wound healing in rats, improved dermis and epidermis regeneration, and had less inflammatory cell infiltration and oedema. This same group of researchers also reported improvement in in vivo re-epithelialization and formation of granulation tissue in a diabetic wound site by applying a metformin and glibenclamide-loaded gelatin/bacterial cellulose nanofibrous template [34].

A collagen/PLGA nanofibrous scaffold membrane was fabricated for sustained release of metformin for wounds associated with diabetes in rat models and the membranes were found to elevate collagen content and effectively promoted wound closure [114]. Another study developed a poly (lactic-co-glycolic acid)/gelatin (PLGA/Gel) nanofibrous scaffold mat for the extended release of liraglutide, an antidiabetic agent known to promote angiogenic activities of endothelial cells. Results of the investigation showed a remarkable decrease in the duration of wound closure, increased blood vessel density, and collagen deposition, all facilitating wound repair [36]. Besides, a nano-configured lipid carrying pioglitazone (an antidiabetic agent) was embedded into a collagen/chitosan composite scaffold template and examined for diabetic wound healing purposes. The scaffolds were non-toxic and in vitro testing in a streptozotocin-induced diabetic wound model enhanced cell growth, an indication of healing, compared to the control [44].

## 6. Conclusions and Future Viewpoints

The skin plays a crucial role by protecting internal organs from harmful microorganisms and other external agents that can disrupt homeostasis. Therefore, it is important to provide it with the utmost care, especially when it is injured. Chronic wounds such as diabetic ulcers often require specialized medical protocol and attention to prevent life-changing complications in affected individuals. Though conventional wound management strategies continue to find use, there are still opportunities for innovative improvements that can optimally accelerate the wound restoration process. Researchers have identified nanofibrous scaffolds as a potential solution to this quest because they have been shown to produce desirable outcomes in the management of diabetic wounds. Thus, further exploration of these novel matrices for minimizing the complications associated with such chronic injuries is beneficial.

We noted that there are several methods of producing nanofibrous scaffolds, with electrospinning being the most widely employed technique because of its simplicity and versatility. It, however, requires high voltage and, as a result, scale-up and industrialization become challenging. Therefore, we consider it important that other flexible and easily scalable fabrication techniques should be explored to produce nanofibrous scaffolds. We also identified that chemicals of plant origin have been shown to effectively enhance wound healing majorly because a single phytochemical usually possesses more than one desirable characteristic that is required for quickening wound recovery. This option, in our opinion, could be cheaper, easily accessible, and pose lesser risks of toxicity compared to utilizing a combination of synthetic bioactive molecules to achieve similar therapeutic outcomes. Thus, encapsulating phytochemicals within nanofibrous scaffold templates could be a way to go for future investigations on discovering optimal and long-lasting solutions to the localized treatment of diabetic ulcers. Although several nanofibers blended with bioactive ingredients exhibited promising results on laboratory animal models, advancing these outcomes to the level of clinical trials on human beings would be beneficial for the assessment of their in vivo performance, therapeutic effects, and possibly commercialization of these scaffolds for diabetic wound repair and recovery.

## Figures and Tables

**Table 1:** Examples of polymer-based nanofibrous scaffold templates and their respective therapeutic activities.

Polymers/Blends	Bioactive Agents	Therapeutic Efficacy	References
Chitosan and polyvinyl alcohol	Zinc oxide	Antioxidant and antibacterial effects and accelerated diabetic wound recovery	[31]
Poly (lactic-co-glycolic acid	Vancomycin, gentamicin, and platelet-derived growth factor	Improved angiogenesis and healing of infected diabetic wounds	[32]
Polycaprolactone and tragacanth gum	Curcumin	Exhibited anti-inflammatory, antioxidant properties, and increased wound closure rate.	[33]
Gelatin and cellulose	Metformin and glibenclamide	Lowered risks of cytotoxicity and improved wound healing	[34]
Polylactide	Doxycycline	Good antibacterialeffect on diabetic wounds	[35]
Poly (lactic-co-glycolic acid and gelatin	Liraglutide	Improved the physical properties of the scaffold template and promoted vascularization on diabetic wound	[36]
Polycaprolactone and gelatin	Amoxicillin and Zinc oxide	Sustained drug release and antibacterial activity	[37]
Chitosan and gelatin	Ferrous oxide	Boosted the antibacterial properties of the scaffold	[38]
Polycaprolactone	Glutathione	Demonstrated anti-inflammatory and antioxidant effects	[39]
Polyvinyl alcohol and sodium alginate	*Calendula officinalis* extract	High wound closure rate and supported cell proliferation	[40]
Silk fibroin	Fenugreek extract	Increased collagen deposition and provided antioxidant benefits	[41]
Collagen/Silk fibroin composite	Fenugreek extract	Enhanced antioxidant properties, improved viability, and proliferation of fibroblasts, which accelerated wound healing	[42]
Poly (lactic-co-glycolic acid), collagen and chitosan	Basic fibroblast growth factor and vascular endothelial growth factor	Promoted angiogenesis, cell proliferation, and prevented scar formation	[43]
Collagen and chitosan	Pioglitazone	Elevated cell growth and rapid wound healing	[44]
Polyvinyl alcohol and polyvinyl acetate	Ciprofloxacin	Antibacterial activity	[45]
Gelatin and polycaprolactone	Aloe Vera extract	Provided anti-inflammatory, antibacterial, and antioxidant effects	[46]
Polyethylene glycol and polycaprolactone	Epidermal growth factor	Enhanced mechanical properties and good healing abilities	[47]
Cellulose and bacterial cellulose	Metformin and glibenclamide	Sustained drug release and anti-inflammatory properties	[34]
Polyvinyl alcohol, sodium alginate, silk fibroin	Asiaticoside	Supplied oxygen to wound and good potential for wound healing	[48]
Gelatin, pluronic-F-127 and polycaprolactone	Bone marrow-mesenchymal stem cell	Promoted angiogenesis, formation of granulation tissue and increased collagen deposits and improved wound healing	[49]

## Data Availability

Data sharing not available.
